# Tailored Approach in Inguinal Hernia Repair – Decision Tree Based on the Guidelines

**DOI:** 10.3389/fsurg.2014.00020

**Published:** 2014-06-20

**Authors:** Ferdinand Köckerling, Christine Schug-Pass

**Affiliations:** ^1^Department of Surgery, Centre for Minimally Invasive Surgery, Vivantes Hospital Berlin, Academic Teaching Hospital of Charité Medical School, Berlin, Germany

**Keywords:** inguinal hernia, endoscopic inguinal hernia repair, Lichtenstein hernia repair, guidelines, plug and patch

## Abstract

The endoscopic procedures TEP and TAPP and the open techniques Lichtenstein, Plug and Patch, and PHS currently represent the gold standard in inguinal hernia repair recommended in the guidelines of the European Hernia Society, the International Endohernia Society, and the European Association of Endoscopic Surgery. Eighty-two percent of experienced hernia surgeons use the “tailored approach,” the differentiated use of the several inguinal hernia repair techniques depending on the findings of the patient, trying to minimize the risks. The following differential therapeutic situations must be distinguished in inguinal hernia repair: unilateral in men, unilateral in women, bilateral, scrotal, after previous pelvic and lower abdominal surgery, no general anesthesia possible, recurrence, and emergency surgery. Evidence-based guidelines and consensus conferences of experts give recommendations for the best approach in the individual situation of a patient. This review tries to summarize the recommendations of the various guidelines and to transfer them into a practical decision tree for the daily work of surgeons performing inguinal hernia repair.

Hernia surgery has become increasingly more complex over the past 20 years due to the introduction of novel endoscopic, but also conventional, techniques. The term “tailored approach” is used to describe the differentiated use of the several different techniques in hernia surgery. Currently, that approach is being used by 82% of experienced hernia surgeons (1). Implementation of the “tailored approach” calls for intense scrutiny as well as widespread experience of the entire field of hernia surgery. The attitude “it’s just a hernia” (2) is a thoroughly outdated view that no longer meets the requirements for successful hernia surgery.

At its 30th annual conference in May 2008 in Seville, the European Hernia Society (EHS) presented for the first time guidelines on treatment of inguinal hernia, going on to publish these in 2009 in the scientific journal *Hernia* (3).

These were followed in 2011 by the guidelines of the International Endohernia Society (IEHS) for endoscopic repair of inguinal hernia (4).

In 2013, the European Association of Endoscopic Surgery (EAES) published the results of a consensus conference of hernia experts (5).

In 2014, the EHS published an update with level 1 study of their guidelines (6).

When the recommendations of the guidelines and of the consensus conference are summarized in terms of the level of evidence (LoE) according to the Oxford criteria, the following differential therapeutic situations must be distinguished in inguinal hernia repair (Figure 1):

1. Primary unilateral inguinal hernia in men.
2. Primary unilateral inguinal hernia in women.
3. Primary bilateral inguinal hernia in men and women.
4. Primary scrotal inguinal hernia.
5. Primary inguinal hernia after previous pelvic and lower abdominal surgery (radical prostatectomy, cystectomy, vascular surgery, and ascites as well as peritoneal dialysis).
6. Primary inguinal hernia in patients who cannot be subjected to general anesthesia because of cardiac or pulmonary risk factors.
7. Recurrent inguinal hernia.
8. Emergency surgery for incarcerated inguinal hernia.

**Figure 1:**
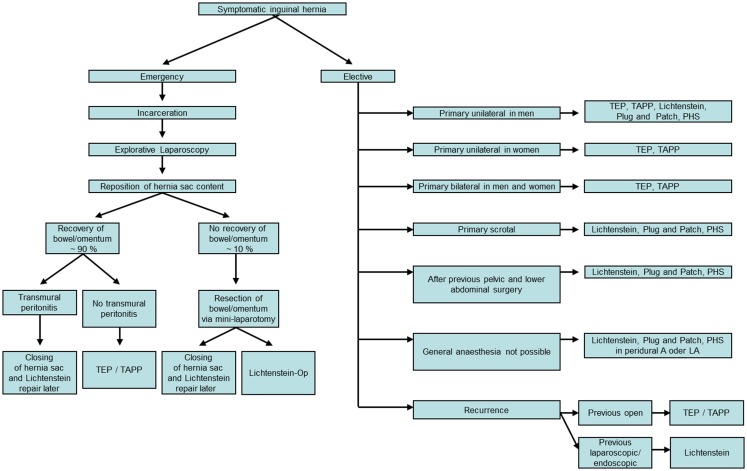
**Inguinal hernia repair based on the guidelines of the European Hernia Society, the International Endohernia Society, and the European Association of Endoscopic Surgery using the “tailored approach**.”

## Primary Unilateral Inguinal Hernia in Men

Based on scientific LoE 1A according to the Oxford criteria, all adult men (>30 years), suffering from symptomatic inguinal hernia should be treated using a mesh procedure regardless of the hernia type (3).

Endoscopic TAPP and TEP procedures as well as the open Lichtenstein technique are the methods of choice for treatment of primary unilateral inguinal hernia (3). PHS and Plug and Patch (mesh plug) result in comparable outcome (recurrence and chronic pain) as the Lichtenstein technique (1–4 years follow-up) (6).

Young, active adult men between 18 and 30 years benefit mostly from endoscopic groin hernia repair because they gain most from early convalescence (5).

The Shouldice technique is the best LoE 1A method, using only a suture and no mesh. But there are two reasons for not using the Shouldice technique: even in expert hands, this mesh-free technique has a 10% recurrence rate after 10 years, and numerous prospective randomized studies and meta-analyses have identified a higher recurrence rate for the open non-mesh techniques (4.4–17%) compared with the open Lichtenstein mesh repair (1–1.4%) (7). Nonetheless, the Shouldice technique continues to be recommended for young adults with a small indirect hernia (LI) (8). However, the long-term findings of the Danish National Hernia Database have identified for men aged 18–30 years who had undergone primary repair of an indirect inguinal hernia a cumulative 5-year recurrence rate of 1.2% following Lichtenstein operations versus 3.9% after suture techniques. Accordingly, the 3.9% recurrence rate seen after suture methods was threefold higher than the 1.2% rate after Lichtenstein operation (*p* = 0.0003) (9).

Therefore, endoscopic TEP and TAPP techniques as well as the open mesh techniques Lichtenstein, Plug and Patch, and PHS currently represent the gold standard for treatment of unilateral, primary inguinal hernia for adult men as from age 18 years. Among the advantages of the endoscopic techniques cited in the guidelines of the EHS are lower rates of wound infections and hematomas as well as earlier resumption of normal, everyday, and working activities compared with the Lichtenstein operation. But on the other hand, the endoscopic procedures take longer and are associated with a higher seroma rate (3).

Besides, the learning curve is longer for the endoscopic techniques.

The IEHS guidelines (4) have identified in meta-analyses, with LoE 1A, that the risk of acute and chronic pain following endoscopic hernia repair is significantly lower than after the open techniques, with and without a mesh (*p* < 0.001). Therefore, the grade A recommendation issued is that preference be given to endoscopic repair of inguinal hernia using a TEP or TAPP technique over the open procedure with and without a mesh, provided that the surgeon has the requisite expertise in endoscopic techniques.

## Primary Unilateral Inguinal Hernia in Women

The Danish Hernia Registry has demonstrated that the risk of recurrence in women following an open technique for primary repair is greater than after an endoscopic method (10), with 38% of reoperations performed because of a recurrent femoral hernia (11). All recurrent femoral hernias occurred in women after previous open repair of inguinal hernias. It must therefore be assumed that the femoral hernia was not diagnosed at the time of primary operation of the inguinal hernia. Because of the diagnostic superiority of endoscopic surgical techniques, the TEP and TAPP are therefore recommended as the repair techniques of choice for women with an inguinal hernia (3, 10). The endoscopic surgical techniques have better intraoperative diagnostic possibilities, while providing an option for optimum subsequent treatment of inguinal and femoral hernia, with demonstrably good results (12, 13).

## Primary Bilateral Inguinal Hernia in Men and Women

The EHS guidelines (3) have identified that the endoscopic technique is the most cost-efficient method for patients who continue to be part of the workforce, with this being particularly true for patients with bilateral hernias (LoE 1B). Likewise, the EAES guidelines (5) recommend the endoscopic technique, in particular, for bilateral inguinal hernia, with equal consideration given to TEP and TAPP. The National Institute of Health and Clinical Excellence (NICE) in England and Wales also recommends endoscopic techniques for bilateral inguinal hernias. In the hands of very experienced endoscopic surgeons, comparable outcomes can be achieved for bilateral as for unilateral inguinal hernias (14). If one views the data in registries, essentially comprising also data for less experienced surgeons, one notes relevant differences in complication rates to the disadvantage of bilateral endoscopic inguinal hernia repair (15, 16). Hence, this too underscores the importance of the surgeon having the requisite expertise.

## Primary Scrotal Inguinal Hernia

In EAES guidelines (5), scrotal hernia is classified as being a complex condition. For scrotal hernia, only highly experienced endoscopic hernia surgeons should opt for a laparoscopic technique (4, 17). The challenge in scrotal hernia is ensuring complete dissection of the large hernia sac from the inguinal canal and scrotum. Failure to remove a large section of the hernia sac will generally result in formation of a persistent seroma (4). Endoscopic control of bleeding during a scrotal hernia repair is also often very difficult when dissecting the hernia sac from the spermatic cord structures. Therefore, there is often a higher incidence of postoperative secondary hemorrhages and hematomas. Accordingly, the EHS guidelines recommend the open mesh techniques (Lichtenstein, Plug and Patch, and PHS) as the techniques of choice for scrotal hernia (3, 6).

## Primary Inguinal Hernia after Previous Pelvic Operations (Radical Prostatectomy, Cystectomy, Vascular Operations, and Ascites as Well as Peritoneal Dialysis)

Faced with these complex situations, the guidelines of the IEHS (4) and the EAES (5) also recommend that only very experienced endoscopic hernia surgeons should opt for a minimally invasive procedure.

Following major lower abdominal and pelvic surgery, the EHS therefore recommends the open mesh techniques (Lichtenstein, Plug and Patch, and PHS) as the preferred techniques (3, 6). The open mesh approach, no doubt, also presents the least risk in the presence of cirrhosis of the liver with ascites or for patients on peritoneal dialysis.

## Primary Inguinal Hernia in Patients Who Cannot be Subjected to General Anesthesia because of Cardiac or Pulmonary Risk Factors

Based on the recommendations of the EHS, the open mesh techniques (Lichtenstein, Plug and Patch, and PHS) under local anesthesia are the preferred techniques when general anesthesia is not possible for patients assigned to ASA III or IV categories because of cardiac or pulmonary risk factors (3, 6). However, data from the Swedish Hernia Registry show that the risk of recurrence after primary inguinal hernia repair is higher under local anesthesia, but that risk is lowest following the Lichtenstein operation (18). Besides, because of the significantly increased risk associated with general anesthesia, there is no alternative to that procedure for this group of patients with symptomatic inguinal hernia.

## Recurrent Inguinal Hernia

In the event of recurrent inguinal hernia following previous open surgery, based on the recommendations of the EHS the endoscopic technique is the technique of choice (grade A), since the operation is performed in an anatomic layer between the peritoneum and the abdominal wall in which no previous dissection had been performed (3, 6). Accordingly, an anterior approach, not touching the preperitoneal space, as in the Lichtenstein operation, should be chosen in the event of a recurrence following previous endoscopic surgical techniques (TEP, TAPP).

The EAES guidelines (5) likewise recommend an endoscopic approach for recurrence following a previous open operation. An endoscopic reoperation after previous TEP or TAPP calls for widespread experience of minimally invasive inguinal hernia surgery and is also classified as constituting a complex situation. As recommendation, a Lichtenstein operation should be performed in such a situation.

## Emergency Surgery for an Incarcerated Inguinal Hernia

In the presence of an incarcerated inguinal hernia, a diagnostic laparoscopy should be performed first of all (4, 5). The incarcerated bowel or greater omentum can then be withdrawn from the hernia sac, if necessary making an incision into the cranial hernia ring. Next, a decision must be taken as to whether parts of the omentum and/or intestines should be resected. In approximately 90% of cases, the data show that this is not necessary as the organs recover after reposition into the abdominal cavity. Then inguinal hernia repair can be carried out using a TEP or TAPP technique. If there is a transmural peritonitis, the hernia sac can be first closed with a suture and the open mesh repair (Lichtenstein, Plug and Patch, and PHS) performed later.

Alternatively, the inguinal hernia can be repaired simultaneously in a different anatomic layer as open mesh repair (Lichtenstein, Plug and Patch, and PHS). If intestinal resection is needed, simultaneous repair of inguinal hernia should be avoided, opting instead for repair at a later stage.

Based on the above, the following decision tree (Figure 1) depicts the differentiated methods of inguinal hernia repair using the “tailored approach.”

## Conflict of Interest Statement

The authors declare that the research was conducted in the absence of any commercial or financial relationships that could be construed as a potential conflict of interest.
